# Complete mitochondrial genome of *Phiaris dolosana* (Lepidoptera: Tortricidae)

**DOI:** 10.1080/23802359.2019.1669083

**Published:** 2019-09-23

**Authors:** Yongyan Li, Wenxu Yang, Jialiang Zhuang, Hao Wei, Xin Liu, Weixing Feng, Haili Yu

**Affiliations:** Shaanxi Key Laboratory for Animal Conservation, Northwest University, Xi’an, Shaanxi, P. R. China

**Keywords:** *Phiaris dolosana*, mitochondrial genome, Tortricidae

## Abstract

We describe the complete mitochondrial genome of Phiaris dolosana. It is 15,562 bp in length, and contains 13 protein-coding genes (PCGs), 22 transfer RNA genes (tRNAs), 2 ribosomal RNA genes (rRNAs), and a 612 bp D-Loop. All PCGs start with ATN codon except for COI gene, which uses CGA as the initiation codon. Nine of 13 PCGs use a typical stop codon of TAA, the rest use incomplete stop codon of T or TA. Phylogenetic analysis of P. dolosana with other 17 leaf rollers is conducted with neighbor-joining method, the result is consistent with the conventional classification.

Genus *Phiaris* Hübner, [1825] 1816 is a member of the family Tortricidae, and closely related to the type-genus of Olethreutinae, *Oletherutes* Hübner, 1822. It is difficult to clearly separate these two genera based on morphological and genital characters, and there are also controversies regarding the classification status of *Phiaris* and some related species (Liu and Li 2002; Li 2011; Nedoshivina 2016; Gilligan et al. 2018). Thus, the main purpose of this study is to provide genetic data for further phylogenetic study. *Phiaris dolosana* (Kennel, 1901) is a widely distributed species in Russian, Japan, and mainland China. The specimen used for the study was collected from Huoditang (33°15′N, 108°15′E), Shaanxi Province, China and preserved in alcohol at Herbarium of Northwest university, China (registered no. NWU.L2018320).

The mitochondrial genes were extracted from the alcohol-soaked muscle tissue with a DNA Extraction Kit (Tiangen Biotech, Beijing, China) and sequenced bidirectionally. The raw data were spliced by Novoplasty-NOVOplasty 3.0 to form a typical circular DNA molecule (Dierckxsens et al. 2017). The complete sequence was annotated with software Geneious 10.0.5 by aligning with reference mitochondrial genome (Kearse et al. 2012). PCGs and tRNAs were compared with the existing sequences in NCBI database to ensure their exact regions. Further, the tRNAscan-SE was used to verify the veracity of tRNA genes (Lowe and Eddy 1997). Phylogenetic analysis was performed with the MEGA 6.0 based on complete mitochondrial genome (Tamura et al. 2013). The circular mitogenomic sequence was deposited in GenBank under the Accession Number MK962620.

The complete mitochondrial genome of *P. dolosana* is 15,562 bp in length, and it is a representative circular DNA molecule containing the genes of 13 protein-coding genes (PCGs), 22 transfer RNAs (tRNAs), 2 ribosomal RNAs (rRNAs) and a 612 bp D-Loop. Nucleotide composition of the complete mitochondrial genome includes A (40.6%), T (40.0%), C (11.7%), G (7.8%), and the content of A + T is 80.5% that is distinctly biased. All PCGs start with ATN codon, except for cytochrome oxidase subunit I (*COI*), with CGA initiation codon. Nine of 13 PCGs have stop codon TAA, and the remaining PCGs contain T or TA stop codon. Seven overlaps at gene junctions were identified – that between *ND1* and *tRNA-Leu* is the longest with 8 bp. Eighteen gene gap regions were also found in a total of 160 bp, and length of the gaps is between 1 and 56 bp. Based on the complete mitochondrial genome from 18 species in Tortricidae, with the outgroup of *Amata formosae* in Erebidae, we generated a neighbor-joining phylogenetic tree (Figure 1), illustrating how *P. dolosana* shows a relationship with some species of Olethreutini than the members of other tribes in Olethreutinae. Such a clustered classification profile is consistent with that generated based on conventional taxonomic methods.

**Figure 1.**
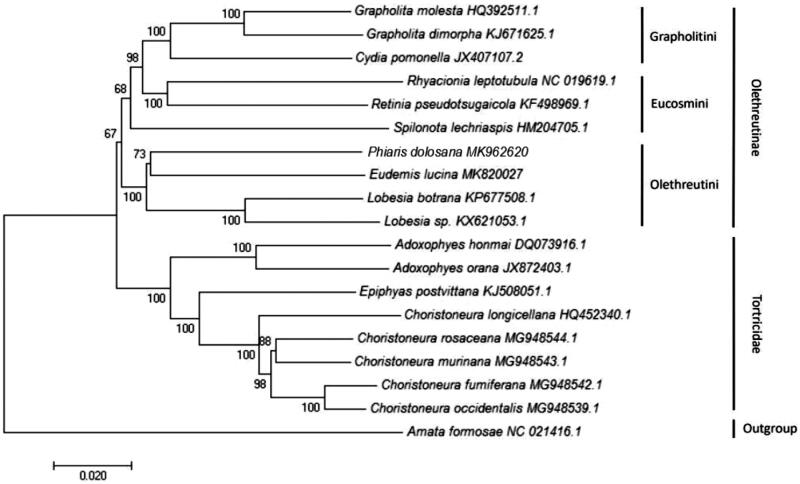
Phylogenetic tree showing the relationship between *Phiaris dolosana* and 17 other leaf rollers based on neighbour-joining method. *Amata formosae* is used as an outgroup. GeneBank accession numbers of each species are also listed.
